# Impact Performance of 3D Printed Spatially Varying Elastomeric Lattices

**DOI:** 10.3390/polym15051178

**Published:** 2023-02-26

**Authors:** Charles M. Dwyer, Jose G. Carrillo, Jose Angel Diosdado De la Peña, Carolyn Carradero Santiago, Eric MacDonald, Jerry Rhinehart, Reed M. Williams, Mark Burhop, Bharat Yelamanchi, Pedro Cortes

**Affiliations:** 1 Advanced Manufacturing Research Center, Youngstown State University, Youngstown, OH 44555, USA; 2 Materials Department, Centro de Investigación Científica de Yucatán, Merida 97205, Mexico; 3 Material Science and Engineering, Youngstown State University, Youngstown, OH 44555, USA; 4 Aerospace and Mechanical Engineering, University of Texas at El Paso, El Paso, TX 79968, USA; 5 Aptiv, 4551 Reseach Pkwy, Warren, OH 44483, USA; 6 Siemens Corporation Corporate Technology, Princeton, NJ 08540, USA

**Keywords:** additive manufacturing, lattices, functionally graded, volumetrically varying, elastomers, impact energy management

## Abstract

Additive manufacturing is catalyzing a new class of volumetrically varying lattice structures in which the dynamic mechanical response can be tailored for a specific application. Simultaneously, a diversity of materials is now available as feedstock including elastomers, which provide high viscoelasticity and increased durability. The combined benefits of complex lattices coupled with elastomers is particularly appealing for anatomy-specific wearable applications such as in athletic or safety equipment. In this study, Siemens’ DARPA TRADES-funded design and geometry-generation software, Mithril, was leveraged to design vertically-graded and uniform lattices, the configurations of which offer varying degrees of stiffness. The designed lattices were fabricated in two elastomers using different additive manufacturing processes: (a) vat photopolymerization (with compliant SIL30 elastomer from Carbon) and (b) thermoplastic material extrusion (with Ultimaker™ TPU filament providing increased stiffness). Both materials provided unique benefits with the SIL30 material offering compliance suitable for lower energy impacts and the Ultimaker™ TPU offering improved protection against higher impact energies. Moreover, a hybrid lattice combination of both materials was evaluated and demonstrated the simultaneous benefits of each, with good performance across a wider range of impact energies. This study explores the design, material, and process space for manufacturing a new class of comfortable, energy-absorbing protective equipment to protect athletes, consumers, soldiers, first responders, and packaged goods.

## 1. Introduction

The complex geometric configurations enabled by additive manufacturing include both regular periodic lattices and irregular stochastic foams. By controlling the distribution of mass throughout a 3D volume, the mechanical properties can be tailored. This includes changes to a customized and intentional anisotropic modulus and strength as well as to energy absorption [1,2,3,4,5]. Furthermore, the density can be either gracefully or abruptly modulated to vary mechanical response from one side to the other within the structure [6,7,8,9,10], in which smooth variation of density is particularly important for avoiding stress concentrations between dissimilar lattice regions. This tunability, along with an increased amount of airflow and cooling, allow lattices to exhibit superior properties to traditional expanded polypropylene helmet foams [11,12]. With the current advances in materials, commercially-available elastomers can now be printed with a diversity of additive manufacturing processes [13,14,15,16,17] Additively manufactured elastomers enable a new class of geometrically complex wearable safety applications, in which soft and durable features enhance both comfort and energy absorption properties.

Parts of common safety padding can be replaced with additively manufactured lattices. For this padding, force-time and displacement-time information is important for analysis and safety improvement verifications. Since one of the main possible use scenarios for lattice protection is in helmets, this data is crucial to examine what may happen to a wearer’s head upon impact. Acceleration applied to the head over a specific period of time is a primary factor in numerous head injury severity measures, such as the Gadd Severity Index [18], Versace Severity Index [19], and NHTSA’s Head Injury Criterion [20]. Research has shown that the risk of traumatic brain injury (TBI) can be decreased with the use of functionally graded lattice structure padding [21,22,23]. This is due to the increased energy absorption capabilities of functionally graded structures, shown to be more efficient in the energy absorbing process due to the differing forces necessary to compress different parts of the structure. Regions of the lattice are specifically designed for low-energy impacts, and others for high-energy impacts. In conjunction with the design, materials such as elastomers have also been demonstrated to provide repeatable energy absorption. Ge et al. wrote that after 100 cycles of compression to a NinjaFlex Kelvin cell lattice, deformation of only 1 mm was observed within seconds of the conclusion of the testing [24]. The sample had risen to full height within 30 min of the testing as well. The rapid return to the original shape is a requirement for wearable padding in contact sports, and consequently, renders additively manufactured elastomeric lattices well suited for the application.

Elastomers have been successfully printed with four of the seven processes defined in the ISO/ASTM taxonomy including [25]: material jetting [26]; vat photopolymerization [27,28]; material extrusion [24,29,30]; and powder bed fusion [31]. These processes have all enabled continuous improvement of the elastomers, and feedstock now includes reliable materials providing high coefficients of restitution and durability as required for commercially available 3D printed athletic equipment (e.g., running shoes, pads). Indeed, lattices have attracted significant attention in the digital context of additive manufacturing, which can create these complex structures more easily than traditional manufacturing. Varying the density of the structures by gradually adjusting the thickness of struts or beams throughout the lattice has been explored for a range of unit cells—each optimized for stiffness or compression performance while reducing the weight of the structure [8,32,33,34,35,36,37]. 

Lattice structures can be stochastic, where the algorithm generates a random structural conformation, or non-stochastic where the structural unit cell is created by tessellation with defined configuration [38]. Similarly, the lattices structures can be divided based on their porosity as open or closed [37]. The performance of lattices is governed by their relative density (ratio between the density of the lattice structure and its solid bulk counterpart), Young’s Modulus, as well as their yield and strength [39]. Lattices can be stretch-dominated which typically provides an initial high strength and stiffness with a failure divided as buckling or stretching, or bending-dominated which show superior strain, a stress plateau with a bending and buckling failure modes [40]. The study of negative Poisson’s ratio unit cells has resulted in the production of lattices with tailored mechanical properties displaying either stretching or bending profile [41]. A recent study on lattices has shown that sheet-triply periodic minimal surface (TPMS) structures exhibit a near stretching-dominated deformation profile while skeletal-TPMS exhibit a bending-dominated behavior [42]. In fact, it was also reported that sheet TPMS structures displayed a superior mechanical performance across ten different unit cell lattice configurations investigated such as Gibson-Ashbey, sheet primitive, and skeletal IWP [42]. Miralbes et al. [43] showed that among six different types of TPMS structures, the Neovious, diamond, and Lidinoid structures were most suitable for safety helmets where high specific energy absorption capabilities are required.

Indeed, a wide range of truss-based unit cell configurations such as cubic, diamond, triangular centered cubic, octahedral, pyramidal, and surface-based (such as spherical shell and gyroid) among many others have been explored in order to evaluate the mechanical performance of the lattices [6,37,44,45,46,47,48,49,50,51,52,53,54,55,56,57,58,59,60,61,62,63]. It has been reported that the behavior of lattices is governed by the unit cell configuration, relative density, and properties of the parent material [64,65,66]. For instance, interpenetrating lattices, which combine the configuration of two different unit cells could result in a system with superior impact performance than their individual counterparts [67]. 

Besides the studies on different configurations, the effect of the printing technology on the resolution and performance of the lattice structures has also been investigated. For instance, Graziosi et al. [49] evaluated the mechanical properties of lattices based on kelvin and BCC configurations using FFF and SLS technologies and found that using an extrusion could result in a higher probability of induced defects with detriments on the mechanical performance, whereas the powder-based process resulted in parts with superior resolution.

Multi-material as well as functionally graded lattices have also been studied to modulate the density in order to optimize both the mechanical, thermal and electromagnetic behavior [34,37,49,54,56,61,68,69,70,71,72]. Brauer and Aukes [72] investigated different methodologies to induce a spatially graded transition on 3D printed materials showing that the best performance can be achieved by a transition process that can provide an equilibrium between the gradual change in material properties and the mechanical bond.

For instance, lattices have been 3D printed for deformable performance of Defense Advanced Research Projects Agency (DARPA) [73,74,75,76,77,78], through their TRAnsformative DESign program, funding research into the representation of ultra-scale structures with many times the level of complexity that can be currently represented in modern CAD software. Siemens, as part of their performance on the TRADES program, have developed a software called Mithril for the programmatic design, analysis, and manufacturing of extreme complexity structures (up to 1012 elements or more). Mithil allows a lattice designer to program a lattice in Python with access to all the usual Python libraries and programming idioms, such as for loops and open-source NumPy libraries. The lattice is stored as this program code (many orders of magnitude more compact than typical CAD representations) and a C/C++ based backend permits the visualization and rapid analysis of that lattice. Programming lattices in this way permits great flexibility in design, enabling easy grading of the size of lattice elements throughout a volume, the manner in which the lattice elements repeat in any direction, and the manner in which the elements are connected.

The Siemens DARPA TRADES team has also developed novel methods for the analysis, optimization, and manufacturability of these highly complex designs. Empirical results obtained in the physical testing at Youngstown State University provided the necessary data for validation of digital models for Siemens DARPA research. As lattices are represented entirely by text-based program code, every part of the representation is effectively a parameter. Optimizing such designs is a simple matter of exposing software parameters of interest to a multi-variable design of experiments and optimization software, such as HEEDS. In this way, designed lattices can be quickly and predictably tuned to provide improved performance for different load cases or use cases [79]. Generative design techniques can also be used to define the material properties under different loads or locations and to produce an equivalent manufacturable lattice [80].

A single Kelvin cell lattice design (a bitruncated cubic honeycomb rod-and-ball lattice) [81] was created with two separate single material systems and one combination of both in order to replace and improve the protection of internal pads within American football helmets [24]. This specific Kelvin cell lattice design has been classified as a bending-dominated structure and shows a robust energy absorption performance when compared to many different unit cell configurations [42]. According to Hawreliak et al. Kelvin cell lattices are approximately isotropic, and their strength is strongly dependent on the thicknesses of their struts [82]. Research performed by Khosroshahi et al. shows that bending dominated structures, like Kelvin cells, are more effective at absorbing impact energies than stretching dominated structures [21]. Kelvin cell lattices are dependent on certain scaling relationships to determine their properties. As relative density increases, the stiffness and strength of a Kelvin cell lattice do not increase as rapidly as that of an Octet-truss structure (scaling constants of 1.60 and 1.63 to 1.77 and 1.88, respectively) [83]. These fundamental properties allow performance predictions for different lattices before testing is done.

In this study, two spatial variations of the Kelvin cell lattice were tested (vertically-graded and non-graded). Two different elastomeric materials were also used to compare their performance at different impact energies. A hybrid sample was also tested, consisting of one of each material lattice layered together. The two materials—Carbon SIL30 elastomer and Ultimaker™ TPU 95A—reside on opposite ends of the stiffness spectrum (Young’s Modulus). This work focuses on the use of elastomer lattices with combinations of non-grading, vertical-grading, and multiple materials in order to investigate structures for absorbing energy under impact conditions. While both materials offered benefits at specific sub-ranges of energy, the multi-material, vertically-graded hybrid lattice provided improved performance in the context of integrating both materials simultaneously under a widened range of impact conditions. Additive manufacturing limits on strut size and material limits at significant infill can be mitigated by combining materials together.

## 2. Materials and Methods

This research effort was focused on creating a benchmark structure to evaluate two different additive manufacturing processes that can fabricate complex, high-resolution lattices with elastomer feedstock. The processes included vat photopolymerization and thermoplastic material extrusion; the fabricated structures were evaluated using repeated low-velocity impact tests to emphasize the relevance in wearable applications in athletics [24].

### 2.1. Geometry Generation of Functionally Graded Lattices

**Figure 1:**
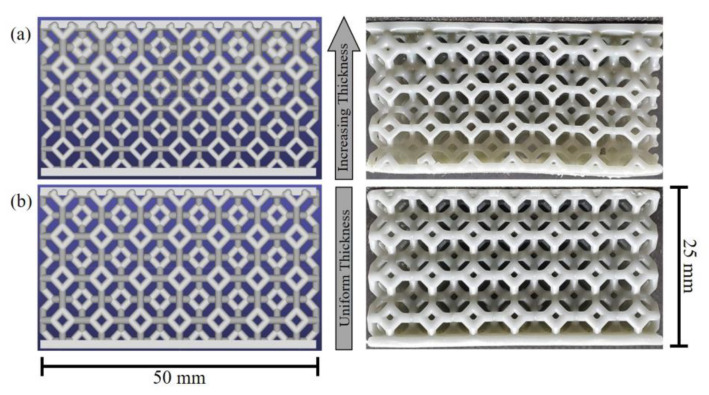
Rendering and photos of the lattice structures investigated in this work based on the Kelvin unit cell (designed in Siemens Mithril software); (**a**) vertically-graded SIL30, and (**b**) non-graded SIL30.

In this study, Siemens’ Mithril software framework was leveraged to design a functionally graded lattice, a configuration which offers spatially stiffness variation depending on the location of the loading. Programming lattice structures rather than designing their geometry directly, as in classic Computer-Aided Design (CAD), offers several advantages. Programming provides a familiar mental model for dealing with the complex (but mathematically well-defined) phenomena that arise from relatively simple rules. A graded Kelvin cell lattice was programmed in Python with the custom Mithril Library. This lattice was chosen for approximately isotropic behavior, especially along the principal axes—making it useful for mitigating impacts that may not be perfectly aligned. It is both geometrically more complex than traditional grid lattices and yet is still based on a classical 3D tessellated structure. Thus, the Kelvin cell lattice design is capable of showcasing the complexity achievable with additive manufacturing. The programmed grading results in 50% thinner struts at the bottom of the vertically-graded lattice. The size of the lattice samples was 50 mm × 50 mm × 25 mm with a lattice cell repetition of 7.07 (5 × $\sqrt{2}$) in x, y, and z, resulting in a bulk sample made up of 7 mm × 7 mm × 7 mm lattice cells (see Figure 1). It is worth mentioning that the vertically-graded parts showed a slightly uneven strut thickness in the horizontal direction due to variability of the actual printing process.

### 2.2. Additive Manufacturing Processes of Elastomeric Structures

Two materials were investigated separately and in combination with lattice designs including both uniform (NG) and vertically-graded (VG) configurations. Table 1 shows the material properties and variations that were selected to explore the effect of modulating both the material and spatial variation within a lattice. As expected, the uniform (non-graded) arrangements have stiffer compression properties in comparison to those with vertical grading. Conversely, the vertical grading provides a transition from thinner struts to gradually thicker struts from top to bottom in the z direction (build direction).

### 2.3. Thermoplastic Material Extrusion of Ultimaker™ TPU

Lattices made from Ultimaker™ TPU (thermoplastic polyurethane) filament were printed at Siemens in Princeton, New Jersey on an Ultimaker™ 3 Extended (UM3x) material extrusion printer (Utrecht, Netherlands), the printer properties and operation parameters of which are as in Table 2. A 0.4 mm (Type AA) nozzle was used. Prints required between six and eight hours at 100% infill density for struts and beams. The UM3x is a Bowden-tube-type printer, so printing soft filaments poses some challenges; however, with some tuning of print speed, no retraction, bridging and coasting settings on, and reducing the fan speed, good results were possible.

### 2.4. Vat Photopolymerization of Carbon™ SIL30 Elastomer

Similar lattices were created using the Carbon™ SIL30 polyurethane elastomer—a soft, comfortable material that is generally highly-compliant and well suited for human contact. The Carbon M2 system is a vat photopolymerization process that projects UV energy from beneath the build to cure the exposed resin. The print speed and light intensity are controlled by a proprietary software from Carbon3D (Redwood City, CA). The printer properties are as shown in Table 3.

### 2.5. Dynamic Impact with High-Speed Video Evaluation

**Figure 2:**
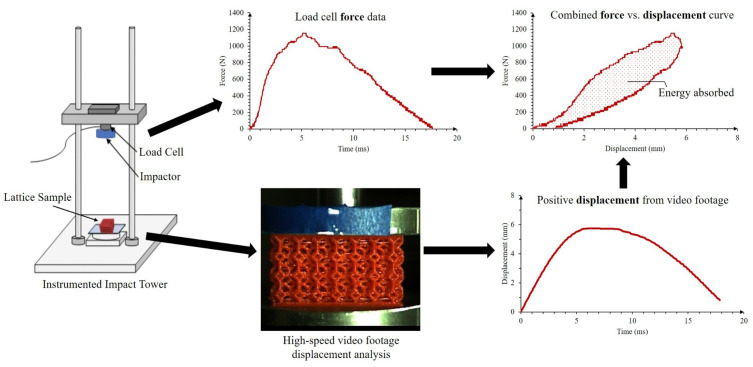
Representation of the impact testing and data analysis. The area enveloped by the force vs. displacement curve is the energy absorbed by the lattice and is shaded above.

Low velocity impact tests were performed on 3D printed elastomeric lattice structures to understand the impact performance for the elastomeric materials. A home-made impact tower with a free-falling flat impactor measuring three inches in diameter was instrumented with a dynamic load cell to collect the force-time relationship of the impact. The impact tests lasted up to 50 milliseconds and were sampled at 200 kHz (for a total of 10,000 data points). The impacts were also recorded with a high-speed Olympus i-Speed 3 camera at 10,000 fps. The recorded videos were analyzed with Tracker software to produce the respective displacement-time plots. The data from the load cell and the high-speed video camera were then combined to generate force-displacement graphs (see Figure 2). The testing consisted of impacting the samples at different heights using a 4.8 kg impactor mass as shown in Figure 2 until the maximum densification of the lattice was observed.

The impact heights were increased in 5 cm increments up to 20 cm, and then every 10 cm, as displayed in Table 4. The impact energy was initially estimated by using Equation (1) and the theoretical impact velocity is calculated using Equation (2).

(1) E=m∗a∗h

(2) E=2∗a∗h

where *E* is the impact energy (J), m is the mass (kg) of the impactor, a is the acceleration (m/s^2^) of the impactor, *h* is the drop height (m) of the impactor.

Velocity and acceleration curves from the video footage displacement were calculated [6]. The specific impact energy, or SIE, was used to compare the performance of the lattices by normalizing impact energy with their masses [88].

### 2.6. Finite Element Simulation

**Figure 3:**
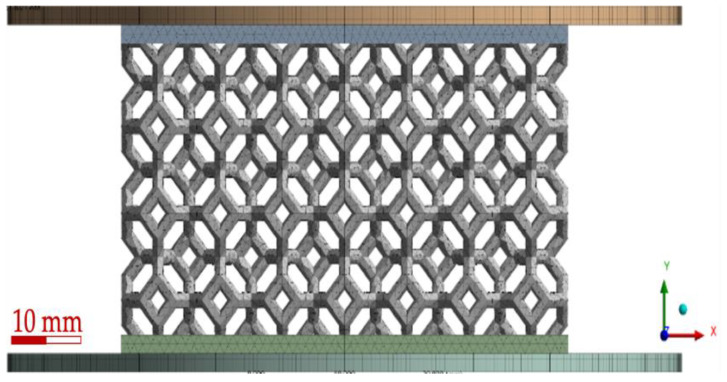
None-graded lattice full mesh with bottom plate and impactor.

The geometry of the non-grade lattice was built in Solidworks^®^ 2020 and imported into ANSYS^®^ Mechanical. Figure 3 shows the full representation of the mesh considered for the non-graded geometry, which includes a bottom plate, the lattice itself, and an impactor. The bottom plate was fully restricted, and the impactor moved only in the y-direction; both were modeled as rigid bodies with structural steel material properties (E = 210 GPa, ρ = 7850 kg/m^3^, υ = 0.3). Lattice material was modeled with Ogden 3rd-order material model, the parameters were fitted using HYPERFIT^®^ 2.181, details of this process can be found in [89]. Frictional contacts were defined between the plates and the lattice with static and dynamic friction coefficients of 0.2 and 0.05, respectively; a similar contact was considered between the lattice struts with coefficients of 1.5 and 1.0 for the static and dynamic coefficients, as described in [89]. A mass point was added to the impactor to match the 4.66 kg of the actual impactor, and standard earth gravity was considered. Initial velocity of 1.1 m/s was assigned to the impactor, which was assumed as the impact velocity from a 50 mm drop height.

## 3. Results and Discussion

Both non-graded (NG) and vertically-graded (VG) configurations of the silicon urethane (SIL30) and the silicon Ultimaker™ (ULTI) were tested under different impact energies. These impact energies were calculated by recording the impact velocity using high-speed video, and subsequently incorporating the kinetic energy equation [88].

### 3.1. Non- and Spatially Graded Lattice Structures

**Figure 4:**
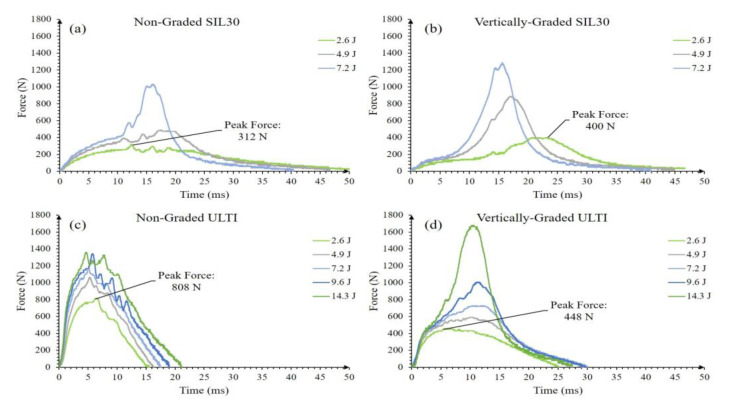
Force versus time of SIL30 and ULTI at different impact energies; (**a**) SIL30-NG (non-graded), (**b**) SIL30-VG (vertically-graded), (**c**) ULTI-NG, and (**d**) ULTI-VG with peak force values labeled for comparison.

SIL30-NG and SIL30-VG lattices were tested at many different impact energies; however, past the impact value of 7.2 J, a sudden force increase is observed as the system reaches the padding threshold capacity. This is a mechanism associated with the high level of densification observed on these relatively soft samples. Therefore, these samples were specifically analyzed for the following impact energies: 2.6, 4.9 and 7.2 Joules (Figure 4a,b). The densification was recorded by a high-speed video camera for further analysis. From Figure 4, the impact events extend to 50 ms, reaching a maximum impact force between 15–25 ms. Increasing the impact energy above 7.2 J resulted in a much higher force due to increased transmission of energy from the impactor through the densified lattice structures, which acts as a solid, and consequently, further energies were not continued. Figure 4b shows that the SIL30-VG showed a superior force profile in comparison to the SIL30-NG. This initial densification is associated with the thin strut regions in the vertically-graded configuration. Figure 4c,d depict the impact response of the ULTI-NG and ULTI-VG supporting higher impact energies than those absorbed by the SIL30 system. However, Figure 4c,d also show that the maximum impact force was reached between 5–13 ms, a shorter-range time than the observed on the SIL30 (15–24 ms). The response of this stiffer material suggests that although the material can be subjected to higher impact forces, the energy absorption performance is limited under low-energy impact events.

**Figure 5:**
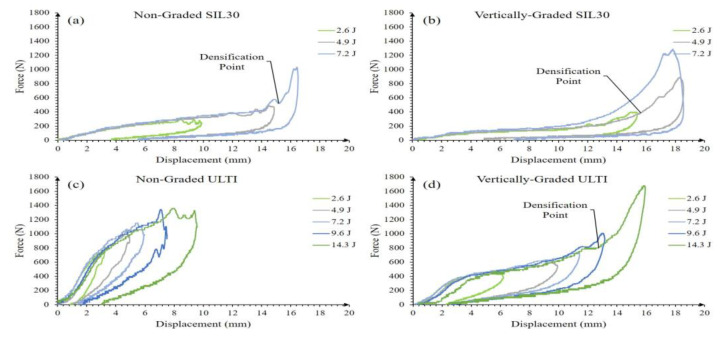
Force versus displacement of SIL30 and ULTI at different impact energies; (**a**) SIL30-NG, (**b**) SIL30-VG, (**c**) ULTI-NG, (**d**) ULTI-VG. Densification points are labeled; however, ULTI-NG does not show densification in these graphs due to its stiffness.

Figure 5 shows the force-displacement response of the SIL30 and ULTI materials based on the uniform- and vertically-graded configurations. The SIL30 displayed an inherently compliant elastomeric profile with a relatively low stiffness, providing improved comfort for wearables (i.e., football helmets) but rendering the material not well suited for high energy impacts due to immediate densification. By comparing the energies imparted to the lattices, it can be observed that the NG system displays a lower displacement than the vertically-graded lattices. For instance, the SIL30-NG non-graded structure was subjected to 2.6 joules and deformed by approximately 10 mm, while the vertically-graded counterpart reached 15 mm. Similarly, the ULTI-VG vertically-graded system shows a larger displacement than the non-graded counterpart. This mechanism seems to be associated with the initial deformation of the thin region struts in the VG configuration. Indeed, the VG structures start with 50% thinner lattice struts at the bottom and thicker struts at the top, resulting in a tailored property and providing increasing stiffness with progressive deformation, in which the initially softer profile of the VG systems promotes earlier densification at lower impact forces.

**Figure 6:**
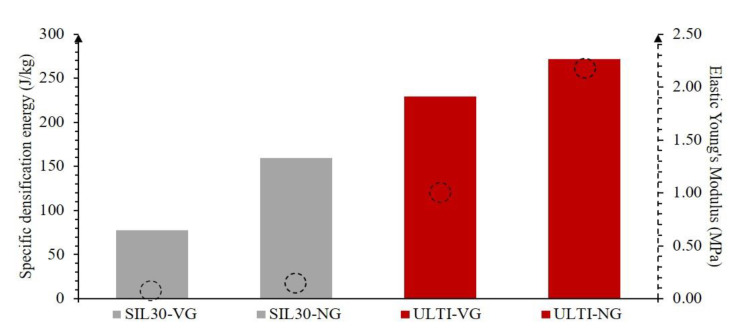
Absorbed energy at the densification point of impact force for the SIL30 and ULTI materials based on the vertically-graded and non-graded configurations, normalized by mass (bars). Elastic Young’s Modulus is shown by the dashed circles and dashed axis on the right side to compare material stiffness as well.

The integration of the area under the curve of the force-displacement plots represents the absorbed energy of the material during the impact event. This integration was performed at the densification point since the analysis at this region allows an equitable comparison across the different configurations. This point refers to the maximum amount of displacement where the lattice system is still capable of effectively absorbing impact energy [42]. After this point, the impact force is fully transmitted throughout the lattice structure, since it is not capable of absorbing any further significant amount of energy, which can result in damage to a protected component. Figure 6 shows the maximum absorbed energy, which corresponds to the densification point, for the four different arrangements investigated, divided by their masses. The energy absorption at the densification points of the vertically-graded configurations is lower than its counterpart on both systems (SIL30-NG and ULTI-NG). The stiffer properties of the ULTI material resulted in specific densification energies 2.9 and 1.7 times higher than the SIL30 for the corresponding lattices. These stiffness values are shown on the right dashed axis by the dashed circles. Young’s modulus is a direct measure of overall elastomeric structure stiffness, and it is shown that the SIL30 material is much more compliant than the ULTI material. Therefore, SIL30 may be better suited for comfort applications and ULTI may be used in a higher energy impact mitigation scenario.

The impact efficiencies of the SIL30 NG, SIL30 VG, ULTI NG, and ULTI VG are at 51.7%, 29.4%, 37.2%, and 33.6%, respectively. It must be noted that while the configurations SIL30 NG, SIL30 VG, and ULTI VG reached the densification point, ULTI NG did not. This indicates that the impact efficiency can go higher. In a work conducted by Ramirez and Gupta [90] on the energy absorption and low velocity behavior of thermoplastic polyurethane foams with densities of 220 kg/m^3^ and 170 kg/m^3^, it was reported that their impact efficiency was 30.49%, and 31.25% at 5 J impact energy and 34.65%, and 30.70% at 7 J impact energy, respectively. The impact efficiency of SIL30 and ULTIM with both NG and VG configurations also seem to be well within the range of the impact efficiency (30%–50%) of the lattices made from multi hit compatible impact attenuator materials such as polyurethane and vinyl nitrile, as studied by Clough et al. [22].

### 3.2. Hybrid Lattice Configuration (Vertically-Graded)

**Figure 7:**
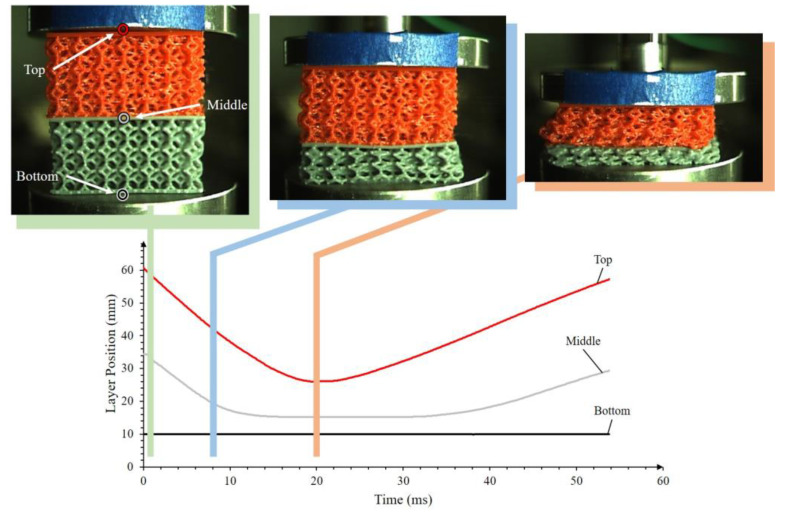
Deformation versus time for intermediate layers of the hybrid approach at a 14.3 J impact. Note that the bottom metal plate thickness is included here and is 10 mm.

A hybrid lattice approach was investigated by stacking the ULTI-VG on top of the SIL30-VG in order to study the dynamic performance of a hybrid structure with two materials based on different impact responses. The vertically-graded configuration was selected since this arrangement allows the analysis of spatially varying stiffness on multi-material arrangements. The stacked samples were subjected to impact energies ranging from 2.6 to 28.3 J as at higher impact energies, the impactor reached the bottom plate through the highly densified sample, and the test was no longer considered valid. Figure 7 shows the deformation of the hybrid lattice subjected to an impact energy of 7.2 J as recorded by the high-speed video camera. This figure provides the amount of deformation and velocity of different sections of the non-uniform lattice. Thinner sections (bottom region of the gray lattice) on the soft SIL30 material densified earlier in the impact event. At around 20 ms, the hybrid system reaches its maximum deformation. The SIL30 layer yielded 19.2 mm of deformation, which was the majority contributor to approximately 68.3% total structure displacement. It was observed that this combined displacement of mixed materials falls between the individual SIL30 and ULTI at 96% and 42%, respectively. After reaching its maximum deflection, the impactor rebounds allowing the sample to recover to the original position.

**Figure 8:**
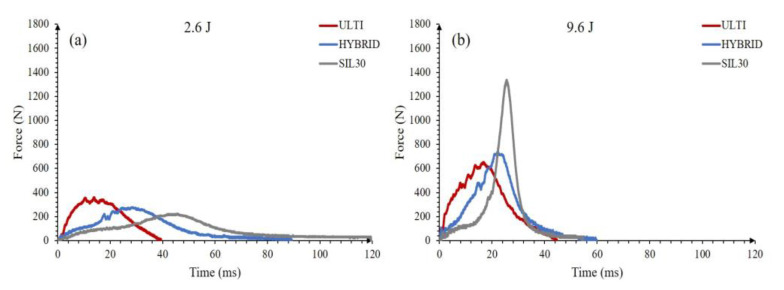
Force response of double stacked vertically-graded configurations; (**a**) 2.6 J, (**b**) 9.6 J.

Low and high impact energies were chosen to evaluate a relatively large range of double-stacked vertically-graded samples’ performances (2.6 J and 9.6 J), as shown in Figure 8. The SIL30-VG deforms more than the ULTI-VG material when tested under low impact conditions. The SIL30-VG displays the maximum force at about 45 ms, a time more than four times larger than that of the ULTI-VG sample. This response is associated with the improved damping nature of the material. The soft profile of the SIL30-VG induces a lower peak force on the load cell when compared with the ULTI-VG. The hybrid lattice shows a response that lies between the SIL30-VG and the ULTI-VG regarding the response time and impact force. A different response is observed under higher impact conditions (9.6 J). The SIL30-VG sample displays a pronounced impact force due to the system reaching the densification threshold. At higher impact energies, the impact forces resulted in higher values when testing the SIL30-VG, since the impactor started striking the bottom plate supporting the samples. These results suggest that the hybrid system offers a blended compromise of both materials. During the impact event, SIL30-VG absorbs most of the initial impact energy, followed by the ULTI-VG when the former has exhausted the energy absorption capabilities. These characteristics suggest that the dynamic performance of lattices can be systematically tailored for targeting specific applications; increasing comfort by reducing stiffness and strut size and increasing high energy absorption capability by increasing stiffness and strut size are examples of this tailor ability.

**Figure 9:**
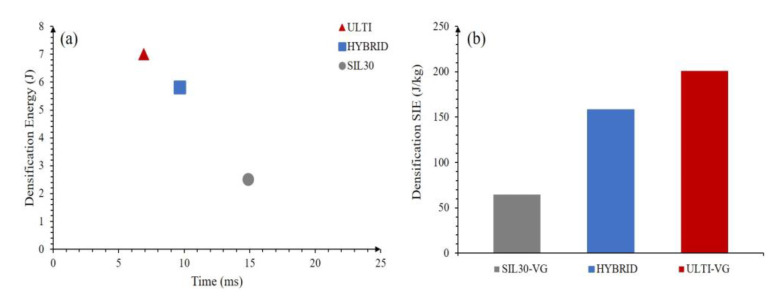
Impact energy response at the densification point for the double stacked vertically-graded systems; (**a**) densification energy, (**b**) densification specific impact energy. The compromise resulting from combining materials is evident here.

Figure 9a shows the maximum absorbed energy reached on all three cases of stacked vertically-graded configurations (SIL30-VG, ULTI-VG and hybrid) at the densification point. It can be observed that the SIL30-VG displays a longer response time due to the softer material and lower overall force profile; however, the soft material lattice has limited energy absorbing capabilities. The densification energy is 3.5 times lower than the ULTI-VG material. In contrast, the hybrid lattice displays a densification time between the SIL30-VG and the ULTI-VG, but with a densification energy considerably higher than that of SIL30-VG. Included in Figure 9b is the normalized comparison of the specific impact energy at the densification point. It is observed that the ULTI-VG lacks such soft impact response but can be capable of achieving impact energies significantly higher than the SIL30-VG. The lattice could be continued to be varied to provide a softer response, but this may require strut dimensions lower than the minimum features sizes possible by the printer and would thus be not printable. On the other hand, the hybrid configuration falls between the ULTI-VG and the SIL30-VG, suggesting that the system can be tailored for shock absorption conditions over a wide range of impact energies.

**Figure 10:**
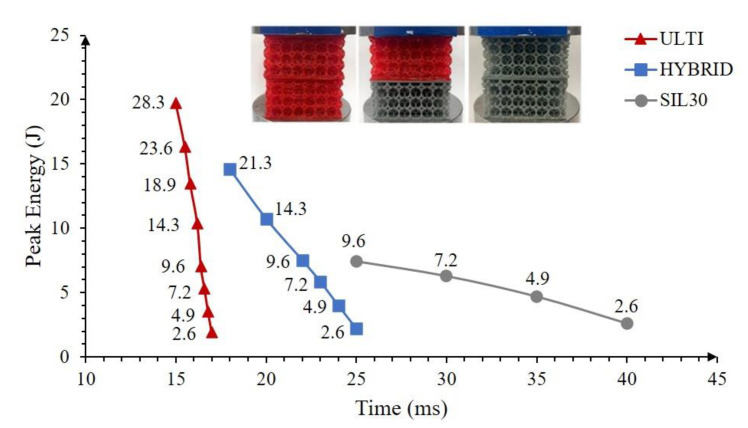
Peak energy absorption from double stacking vertically-graded systems at different impact energies.

Figure 10 shows the maximum impact response for all the vertically-graded groups tested in this study. As it was observed on the impact response at the densification point, the SIL30-VG is the system that sustained the longest response time on every impact event (up to 9.6 J) and thus reduced the maximum deceleration. The system shows an impact time of more than twice the ULTI-VG time when impacted at 2.6 J. In contrast, the stiffer ULTI-VG material showed a maximum higher energy absorption performance almost three times higher than the SIL30-VG. As a compromise between the two systems, the energy and time response of the hybrid stacking falls between that which was recorded on its constituents. This hybrid combination is therefore well suited to be used as impact protection in equipment like helmets. In this scenario, the ULTI-VG acts as the shock absorbing phase to mitigate the high energy from the impact, and the SIL30-VG as the damping component to increase the softness of the response around the head.

**Figure 11:**
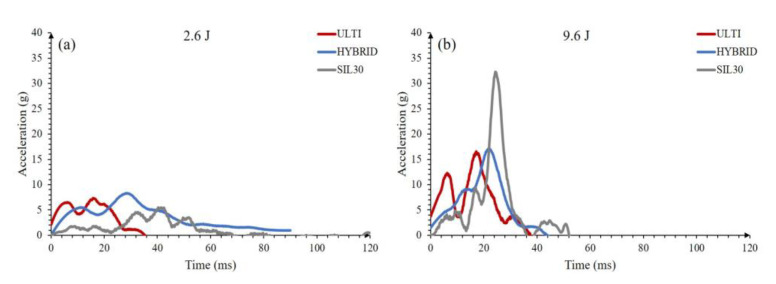
Acceleration response of all three double-stacked vertically-graded configurations; (**a**) 2.6 J, (**b**) 9.6 J.

An acceleration analysis on the hybrid vertically-graded lattice and the constituent counterparts were performed under low (2.6 J) and high (9.6 J) impact energies (see Figure 11). Under both impact energies, the hybrid lattice was able to reduce the velocity change more safely throughout the impact as explained by the following reasoning. Under the impact energy of 2.6 J, the hybrid system lengthened the impact time to more than three times longer than that of ULTI-VG. Also, under the impact energy of 9.6 J, the hybrid configuration displayed a significantly lower peak acceleration than the SIL30-VG, and a slightly longer impact time than the ULTI-VG. Therefore, the hybrid lattice exhibited a more ideal acceleration profile, consisting of both a longer impact time and a lower peak acceleration value than what was observed on either ULTI-VG or SIL30-VG. This combination of materials leads to property compromises that yield good results in many aspects, rather than mechanical outcomes in only one aspect.

The incorporation of geometric- and material-grading within lattices can provide users with an increased level of tunability of the dynamic performance compared to what can be achieved on a single material and uniform system. This strategy may enable designs that provide a comfortable level of impact reduction for lower-energy impacts while absorbing high-energy impacts and reducing the risk of injury (i.e., helmet for head protection). This result shows promise for the future of additive manufacturing of protective equipment. Future work will investigate the possibility to more comprehensively understand the design space including: (a) polymer welding of the two material structures to create a single combined system with embedded sensors and microelectronics, (b) different grading strategies between the two materials for further optimization (including side-grading for possible lateral energy deflection), (c) finite element analysis of the samples to understand performance in ideal scenarios, and (d) a wider range of material choices including Rebound resin from Form Labs (available commercially in the New Balance 3D printed shoe), NinjaTek’s NinjaFlex, and EOS TPE 300.

**Figure 12:**
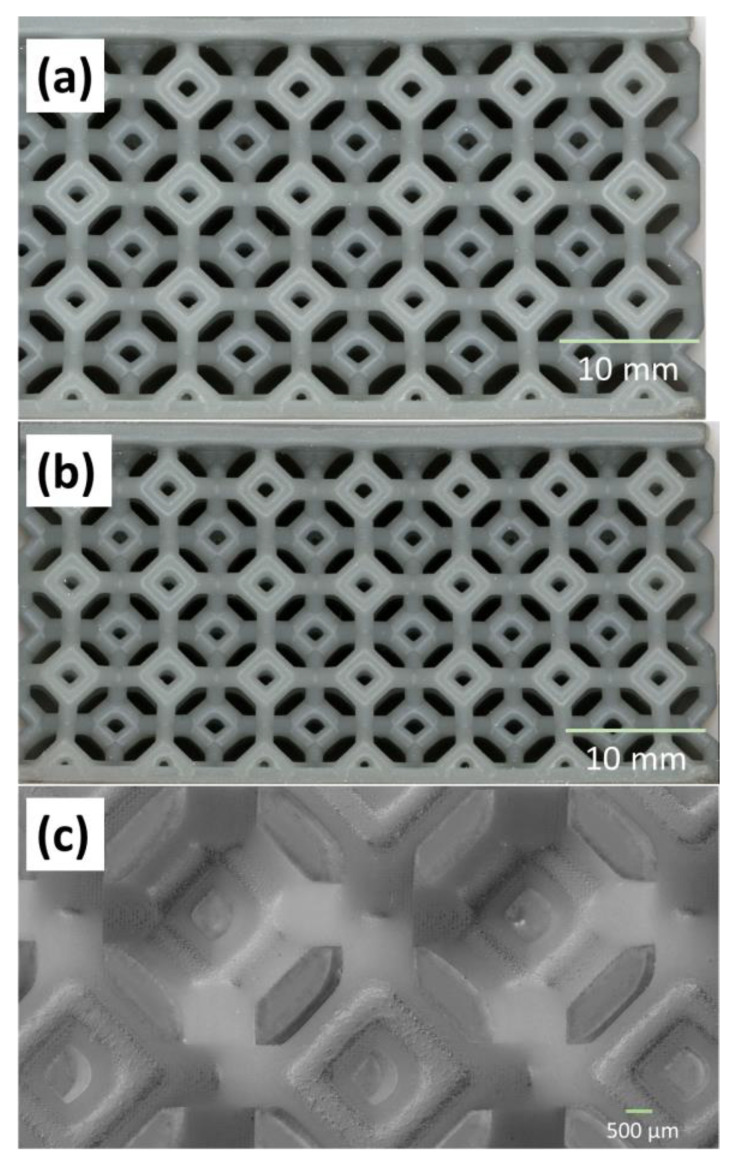
Micrographs of the SIL30 sample (**a**) before impact, (**b**) after impact and (**c**) high magnification view post impact.

The effect of impact on the lattices was performed using a VHX7000 optical/SEM microscope. Figure 12a shows the low magnification micrograph of the SIL30 sample as printed (before impact). The figure displays a high-resolution build quality of all the struts. Figure 12b shows the micrograph of the same sample post impact at 7.2 J, where no visible damage to the sample was observed. To corroborate this finding, a higher magnification analysis was carried out on the struts as seen in Figure 12c, where no fracture or deterioration was observed. Similar results were observed in all the other test samples as well.

### 3.3. Finite Element Analysis Results

**Figure 13:**
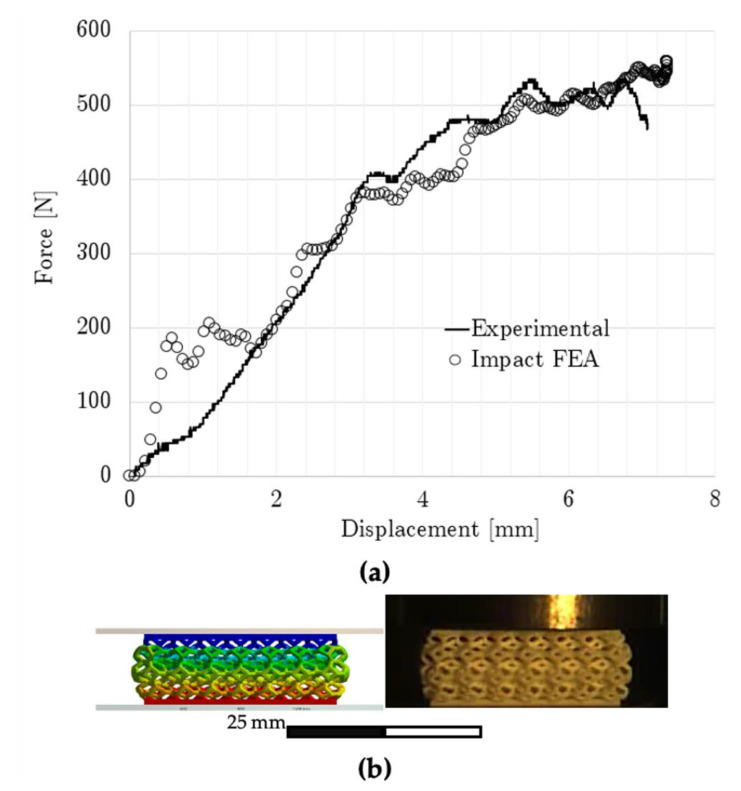
Non-graded lattice numerical results: (**a**) impact force–displacement curve comparison and (**b**) maximum compression under simple impact.

Figure 13a shows the modeled and experimental force–displacement relationships up to the maximum compression reached by the non-graded lattice with simple impact. The maximum compression is displayed in Figure 13b, which contains both experimental and modeling results. By integrating Figure 12a, the absorbed energy of the non-graded lattice differed only by 2% at 0.267 mm/mm between the experimental and numerical results. Also, the maximum displacement was 7.07 mm and 7.35 mm for the experimental and numerical results, respectively, while the maximum experimental force was 534.85 N and the numerical force was 558.84 N.

## 4. Conclusions

Two elastomeric lattice materials (ULTI and SIL30) based on two configurational arrangements (non-graded, NG, and vertically-graded, VG) were studied under a range of impact conditions. Further dynamic evaluations were performed on a hybrid system composed of a combined ULTI-VG/SIL30-VG lattice arrangement. All of the systems investigated were based on a single lattice design to normalize the comparison of materials and functional grading a Kelvin cell lattice configuration, which was created in the Mithril software developed by Siemens and DARPA. The hybrid configuration seems to represent an attractive configuration to be used as an impact material for personal protective equipment like helmets. Here, the ULTI material component of the hybrid structure acts as the shock absorbing phase, and the SIL30 as the damping component. Material and density variations in lattices can yield structures with tailored impact performance for specific applications. This customizability strategy allows the creation of combinations that can deliver high energy impact absorbing capabilities, while also providing sufficient comfort for wearable structures that have dynamic damping properties. The high degree of tailor ability of graded lattices should continue to yield interesting results into the future. The following points summarize the technical aspects of the current study.

- The vertical-graded system displayed larger displacement than its non-graded counterpart during the impact events, a direct result of the 50% thinner struts at the bottom of the VG samples. This softer profile on the vertically-graded configuration resulted in an earlier densification. It was also shown that the ULTI material displayed a significantly stiffer impact response when compared to the SIL30 material. Therefore, the densification energy of the ULTI was about two times higher than the SIL30, suggesting applications in more extreme impact conditions.
- Impact analysis on stacked vertically-graded lattices showed that although the SIL30-VG was not capable of supporting impact energies higher than 9.6 J, it had a more lengthy response than the ULTI-VG material under low energy impacts. In contrast, the ULTI-VG was able to withstand impact energies as high as 28.3 J but absorbed the energy over a much shorter period of time resulting in higher forces.
- The incorporation of a hybrid lattice resulted in a structure that displayed benefits of both materials. Observed were a densification time (10 ms) falling between that of the SIL30-VG (15 ms) and the ULTI-VG (~7 ms), and a densification energy considerably larger than that of SIL30-VG. In cases, in which the minimum feature size is reached for a given printer, including a second soft material can provide a wider range of impact energy management.
- Acceleration analysis on the impacted materials showed that the hybrid lattice was able to effectively manage impacts of varying energies by limiting the force measured on the impactor and by lengthening the total impact time. During the 2.6 J impact, the hybrid and SIL30-VG samples recorded impact times in excess of 60 ms, while the ULTI-VG lattice impact lasted less than 36 ms. Meanwhile, during the 9.6 J impact, the load cell experienced up to 17 g of acceleration with the hybrid and ULTI-VG samples, while the SIL30-VG material allowed the impactor to reach over 31 g. Therefore, unlike the hybrid system, the individual samples were not able to employ both strategies at once for both high energy impacts and low energy impacts.
- The non-graded lattice FEA results resembled the experimental data, the displacement and force showed good agreement. A similar modeling procedure can be used to numerically evaluate additional spatially graded lattice configurations.

## Figures and Tables

**Table 1:** Metamaterial quasi-static characteristics.

Label	Printer Process	Spatial Variation	Structural Density * kg/m^3^	Material Density kg/m^3^	Volume Fraction %	Structural Stiffness MPa	Material Stiffness ** MPa (Hardness S)
SIL30 Non-Graded	Vat Photo Polymerization	Uniform	367	1070	34.3	0.14	1.54 (35 Shore A)
SIL30 Vert.-Graded	Vat Photo Polymerization	Vertical	310	1070	28.9	0.07	1.54 (35 Shore A)
ULTI Non-Graded	Material Extrusion	Uniform	284	1220	23.2	2.17	39.1 (95 Shore A)
ULTI Vert.-Graded	Material Extrusion	Vertical	248	1220	20.4	1.00	39.1 (95 Shore A)
Hybrid Vert.-Graded	Both	Vertical	277	N/A	24.6	0.13	N/A

* Density of the lattice including voids, ** [84,85].

**Table 2:** Ultimaker™ 3 printer properties and operation parameters [86].

Properties/Parameters	Value
Layer resolution	200–20 µm
XYZ resolution	6.9, 6.9, 2.5 µm
Build speed	<24 mm^3^/s
Nozzle temperature	180–280 ℃
Build plate temperature	20–140 ℃

**Table 3:** Carbon M2 printer properties [87].

Properties/Parameters	Value
Pixel size (XY resolution)	75 µm
Slice thickness	100 µm
Wall thickness	2.5 mm
Unsupported angle	40°
Overhangs	1 mm

**Table 4:** Height, energy, and velocity of the experimental impact events.

Height (cm)	Impact Energy (J)	Velocity (m/s)
5	2.6	1.04
10	4.9	1.43
15	7.2	1.74
20	9.6	2.00
30	14.3	2.44
40	18.9	2.81
50	23.6	3.14
60	28.3	3.44

## Data Availability

The data presented in this study are available on request from the corresponding author.
